# Retrospect and Prospect

**Published:** 1892-12

**Authors:** 


### Editorial, Etc.

RETROSPECT AND PROSPECT.—Everything in this world has a beginning and an end. The current (December) number marks one year's connection of the writer with the JOURNAL, which in that time has spoken on a great variety of subjects. For the coming year it is expected that additions will be made in the interest and value of the contents, and that the features will be so shaped as to be an aid to the dental practitioner. While during the year the JOURNAL has been made up partly of selections from other periodicals with only occasional original articles, and has been issued often three months behind date, yet it is gratifying to know that the contributions have been of general interest, and that the publishers have determined not to let the issue get behind again.

In the future through reviews of current periodicals, subscribers of the JOURNAL may learn their contents and thus be enabled to select judiciously additions to their libraries.

For years THE AMERICAN JOURNAL OF DENTAL SCIENCE maintained a foremost place, and was the medium chosen by the best minds of the dental profession for the presentation of their papers. Why cannot the brightest talent be enlisted again in its behalf? Original contributions are solicited, and authors will be supplied with copies of the JOURNAL.

Some of the original topics discussed in the numbers of the JOURNAL published this year have been: "Concentration as a Principle of Success"; "Dental Legislation" together with the dental laws of Maryland, of Texas and of the District of Columbia; "The Conservation of Eyesight in Dental Operations"; "Opposition to a New Dental College"; "How We Treat and Fill the Canals of Abscessed Teeth Successfully;" "Bridge and Crown Work"; "Dentists and the Census Bill;" "The Dentist a Teacher"; "Culture and Professional Success."

Dental Society proceedings, papers published in other periodicals in this country or elsewhere, have also been reprinted or condensed. R. G.

---

